# Cross-sectional analysis of blood leukocyte responsiveness to interleukin-10 and interleukin-6 across age and physical activity level

**DOI:** 10.1515/teb-2024-0027

**Published:** 2024-11-14

**Authors:** Hashim Islam, Jordan Boultbee, Garett S. Jackson, Alice L. Mui, Jonathan P. Little

**Affiliations:** School of Health and Exercise Sciences, University of British Columbia Okanagan, Kelowna, BC, Canada; Centre for Chronic Disease Prevention and Management, University of British Columbia Okanagan, Kelowna, BC, Canada; Department of Surgery, University of British Columbia, Vancouver, BC, Canada; Department of Biochemistry and Molecular Biology, University of British Columbia, Vancouver, BC, Canada

**Keywords:** chronic inflammation, monocytes, lymphocytes, STAT3, inflammaging, exercise

## Abstract

**Objectives:**

To determine how the anti-inflammatory actions of interleukin-10 (IL-10) and IL-6 differ across age and physical activity levels.

**Methods:**

Using a cross-sectional design, fasted blood samples were obtained from younger physically inactive (YI: n=10, age: 22.7 ± 3.7 years, BMI: 24.8 ± 4.8 kg/m^2^, <150 min of weekly moderate-to-vigorous physical activity [MVPA]), younger highly active (YA: n=11 varsity cross country running athletes, 20.7 ± 2.7 years, 21.1 ± 1.8 kg/m^2^, >300 min of weekly MVPA), and older highly active (OA: 12, 56.0 ± 10.3 years, 22.8 ± 3.2 kg/m^2^, >300 min of weekly MVPA) individuals and analyzed for leukocyte counts, IL-10 and IL-6-related signaling, and cytokine secretion *ex vivo.*

**Results:**

Total white blood cells and monocytes were similar between groups (p=0.8) but YA and OA had lower lymphocyte counts than YI (p<0.01). The ability of IL-10 (1 ng/mL) to phosphorylate signal transducer and activator of transcription 3 (STAT3) in CD14 monocytes was greater in YA vs. YI (p<0.03) despite YA having lower IL-10 receptor expression (p<0.01). IL-6 (10 ng/mL) mediated STAT3 phosphorylation in CD4 lymphocytes was higher in OA compared YI (p<0.01), with a similar tendency observed for YA vs. YI (p=0.08). Despite enhanced responsiveness of STAT3 to IL-10/6 in active individuals, the ability of IL-10/6 to inhibit tumor necrosis factor-alpha (TNF-⍺) secretion from lipopolysaccharide-stimulated whole-blood was similar between groups.

**Conclusions:**

Highly active younger and older individuals demonstrate enhanced IL-10- and IL-6-mediated activation of immune cell STAT3. Although the ability of IL-10/6 to inhibit TNF-⍺ secretion appeared unimpacted by activity level, anti-inflammatory cytokine actions were preserved in older active individuals.

## Introduction

Inflammatory responses are critical for combatting illnesses, maintaining cellular homeostasis, and promoting tissue repair following injury, but unresolved sterile inflammation – commonly referred to as “chronic inflammation” – contributes to various chronic diseases [1, 2]. Regular exercise is a known powerful anti-inflammatory stimulus [3]. A major mediating mechanism is the release of anti-inflammatory cytokines from contracting muscle (e.g., interleukin [IL-6]), which stimulates the production of anti-inflammatory cytokines from circulating immune cells (e.g., IL-10), thereby counteracting the release of pro-inflammatory mediators that drive chronic inflammation [4, 5]. The purported existence of this “IL-6 cascade” has led to the widespread practice of quantifying the anti-inflammatory effects of exercise via measurement of circulating cytokine concentrations [6, 7].

Although reflective of changes in the crude systemic inflammatory milieu, circulating cytokine concentrations do not provide any insight into the cellular source/target of cytokine secretion/action nor do they capture exercise-induced changes in cellular inflammatory processes [8]. We have recently demonstrated that an acute bout of higher intensity exercise can induce a transient hyporesponsiveness to the anti-inflammatory actions of IL-10 (direct) and IL-6 (indirect) in blood leukocytes – as reflected by their ability to activate signal transducer and activator of transcription 3 (STAT3) and inhibit tumour necrosis factor-alpha (TNF-⍺) secretion in response to lipopolysaccharide (LPS) [9]. We have previously reported similar observations of attenuated anti-inflammatory cytokine action following 2 weeks of exercise training in individuals with obesity [10]. Importantly, in both these cases, circulating cytokine concentrations dissociated from changes in cellular cytokine action, accentuating the need to look beyond circulating cytokine concentrations when evaluating the immunomodulatory effects of exercise. Relatedly, whether the hyporesponsiveness to anti-inflammatory cytokine is a transient phenomenon observed after acute exercise and short-term training, or apparent under chronically high levels of physical activity is not known.

**Figure 1:**
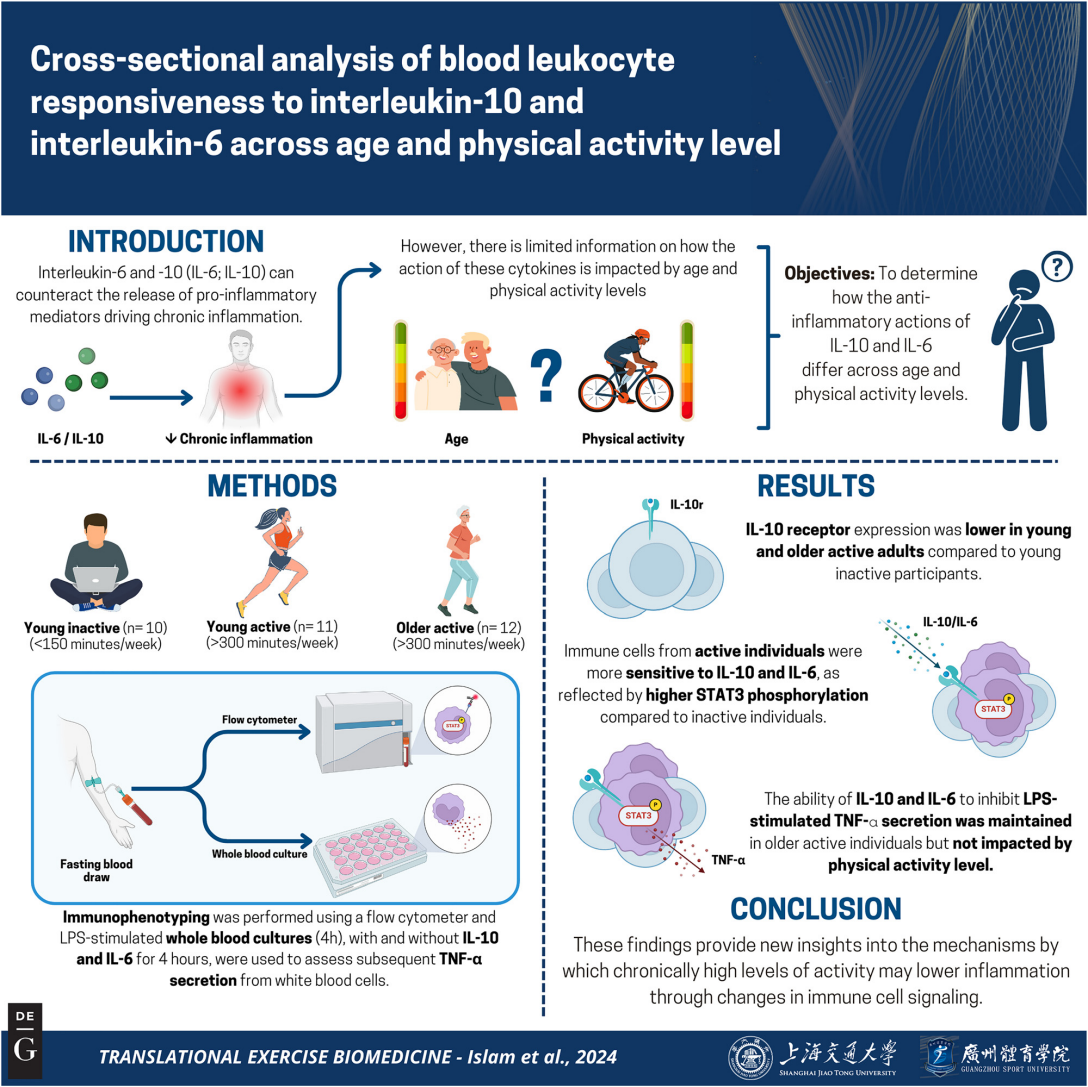
Graphical representation of this study. Key points: (1) A major mechanism by which exercise protects against chronic disease is by lowering chronic inflammation; (2) Much of what we know about the anti-inflammatory effects of exercise is derived from circulating cytokine concentrations, which may not reflect inflammatory processes at the cellular level; (3) Here we show that the ability of interleukin (IL)-10 and IL-6 to activate anti-inflammatory signaling in immune cells is enhanced in highly active individuals compared to those who are inactive – an effect that is apparent in both younger and older active individuals. These observations advance our understanding of the inflammation-lowering effects of physical activity. Figure created with BioRender.

The primary objective of this exploratory cross-sectional study was to determine how blood leukocyte responsiveness to IL-10 and IL-6 – two cytokines most heavily implicated in the anti-inflammatory effects of exercise [8] – is altered across individuals at opposite ends of the physical activity spectrum. Given the known impacts of aging on the immune system [11], we also wanted to determine whether chronically high levels of physical activity were able to preserve IL-10/6 action in highly active older individuals compared to their younger counterparts. To test these objectives, we compared the ability of *ex vivo* IL-10/6 stimulation to induce STAT3 phosphorylation in circulating monocytes and lymphocytes from inactive younger individuals (18–30 years of age performing <150 min of weekly activity), younger highly active individuals (18–30-year-old varsity cross-country athletes performing >300 min of weekly activity), and older highly active individuals (>40-year-old adults performing >300 min of weekly activity). The ability of IL-10 and IL-6 to inhibit TNF-⍺ secretion in LPS-stimulated whole-blood was also determined in parallel. The summary of this article is presented in Figure 1.

## Methods

### Participants

Participants were recruited from Kelowna, BC and surrounding areas as part of a larger cross-sectional study investigating IL-10 function across the health span. Participants were included if they were between: 1) 18–79 years old and had a BMI within 18.5 and 40.0 kg/m^2^. Participants were excluded if they: 1) had been sick in the previous 3 weeks, 2) were current smokers, 3) had a history of heart attack, stroke, coronary/peripheral artery disease, revascularization surgery, cancer, COPD, 4) were using of immunomodulating medications, 5) were currently following an extreme diet (e.g., ketogenic, intermittent fasting), 6) were unable to travel to and from the university for blood sampling, 7) were unable to follow the controlled diet (∼8-h overnight fasted) and physical activity (no structured exercise for 24 h before lab visit) instructions required for the study, and 8) were currently pregnant or planning to become pregnant during the study (if female). Once deemed eligible, participants were categorized into the following groups: younger inactive (YI: 18–30 years of age not currently meeting physical activity guidelines), younger active (YA: 18–30 years of age currently engaged in competitive endurance training), older active (OA: ≥40 years of age currently engaged in competitive endurance training). Informed consent was obtained from all participants prior to data collection in accordance with the Declaration of Helsinki. All data collection was performed at The University of British Columbia (Okanagan Campus). The study was approved by the institutional clinical research ethics board (approval number: H20-00240).

### Experimental visit

All participants completed a single laboratory visit in an overnight fasted state (≥8 h) having refrained from physical activity for 24 h leading up to the visit. Anthropometrics (height, weight, waist circumference), resting heart rate, and blood pressure measurements were made upon arrival. A venous blood sample was then obtained from an antecubital vein using a 21-gauge butterfly needle into EDTA vacutainer tubes. Blood was processed within 15 min for the determination of complete blood counts using an automated hematology analyzer (Beckman Coulter DxH 520) or for the measurement of intracellular signaling and cytokine secretion (see below). Participants were also asked to complete the International Physical Activity Questionnaire Short Form questionnaire to quantify weekly physical activity levels and detailed training diaries were requested from the active groups to further characterize their physical activity.

### Flow cytometry

The detailed flow cytometry protocol and gating strategy have been previously published [12]. Briefly, whole blood was stimulated with recombinant human IL-10 (1 and 100 ng/mL; 130-098-448) or human IL-6 (0.1 and 10 ng/mL; 130-095-365) for 12 min and stained with CD14 (130-110-524), CD4 (130-113-250), CD210 (130-127-892) and CD126 (130-128-359) antibodies (1:100 dilution for all). Recombinant cytokines and antibodies were purchased from Miltenyi Biotec. Samples were then lysed and fixed (BD Phosflow, BD Biosciences), washed twice (10 min at 300 g) with BD Pharmingen Stain Buffer with FBS (BD biosciences), and permeabilized (30 min on ice using BD Phosflow Perm Buffer III, BD Biosciences). Intracellular staining for pSTAT3 (1:20 dilution, Alexa Fluor 488 mouse anti-STAT3 antibody pY705, BD Biosciences) was performed for 30 min in the dark. Samples were analyzed using a flow cytometer (Cytoflex S, Beckman Coulter, Indianapolis, USA). The median fluorescent intensity (MFI) of pSTAT3, CD210 (IL-10 receptor), and CD126 (IL-6 receptor) in CD14+ monocytes and CD4+ lymphocytes was measured (≥10,000 events for each cell type). Fluorescence minus one control tests were used to determine gating on positive and negative populations, and compensation was performed to account for spectral overlap.

### Whole blood cultures

TNF-α production was determined in LPS-stimulated whole-blood cultures as described previously [12]. Briefly, fresh EDTA blood was diluted 10-fold with serum-free RPMI media (Sigma Aldrich, MA, USA) containing 5 mM glucose, penicillin (50 ug/mL) and streptomycin (50 ug/mL). Whole blood was seeded into a 24-well culture plate and stimulated with 1 ng/mL LPS (L5418; Sigma) in the presence or absence of 1, 2.5, 5 and 10 ng/mL of recombinant human IL-10 (130-098-448) or IL-6 (130-095-365) for 4 h at 37 °C and 5 % CO_2._ Following incubation, the culture supernatants were collected and stored at −80 °C until analyses. Samples were batch analyzed according to manufacturer’s instructions for the measurement of secreted TNF-α via ELISA (Human TNF-α DuoSet, R&D Systems, MN, USA).

### Statistical analyses

Data were analyzed using GraphPad Prism Version 10. Participant characteristics, anthropometrics, complete blood counts, weekly physical activity, cytokine receptor expression, and STAT3 phosphorylation were analyzed via separate one-way analyses of variance (ANOVA). Mixed effects models with group and concentration as fixed factors and participant as a random factor were used to analyze IL-10 and IL-6 mediated inhibition of TNF-α secretion from LPS-stimulated whole blood. Significant main effects or interactions were explored using Tukey post hoc tests. Significance was set at p≤0.05. Data are reported as means ± standard deviation (SD).

## Results

### Participant characteristics

Participant characteristics are shown in Table 1. A total of 10 YI, 11 YA, and 12 OA participants were included in this cross-sectional study. All of the individuals in the YA group were active members of the UBCO varsity cross country team. Most of the individuals in the OA were master’s athletes currently competing in endurance events (e.g., half-marathon, marathon, Ironman) at the regional and/or provincial level. Most of the individuals in the OA group were middle-aged (i.e., between 40 and 60 years old) with three individuals in this group being classified as elderly (i.e., ≥65 years). Significant between-group differences were apparent for age (OA vs. both YI and YA, p<0.01), BMI (YA vs. YI, p=0.04), RHR (YA and OA vs. YI, p<0.01), and vigorous activity (YA and OA vs. YI, p<0.01). Although total white blood cell counts were not different between groups (p=0.80), both the YA (p=0.04) and OA (p<0.01) groups had lower lymphocyte counts than the YI group. The YA and OA groups (both p<0.01) reported engaging in more vigorous activity per week than the YI group, though weekly moderate activity was not significantly different between groups (p=0.11). Weekly training diaries obtained from a subset of the YA (n=7) and OA (n=10) groups indicated that both were predominantly composed of runners, though the OA group also engaged in cycling, swimming, and resistance training.

**Table 1:** Participant characteristics.

	YI (n=10)	YA (n*=*11)	OA (n=12)
Sex, M/F	5/5	5/6	6/6
Age, years	22.7 ± 3.7	20.7 ± 2.7	56.0 ± 10.3**
BMI, kg/m^2^	24.8 ± 4.8	21.1 ± 1.8*	22.8 ± 3.2
WC, in	33.6 ± 5.4	30.1 ± 2.3	32.7 ± 3.4
SBP/DBP, mmHg	116/69 ± 12/9	115/66 ± 9/9	120/74 ± 8/7
RHR, bpm	67 ± 5	57 ± 5*	56 ± 8*
Total white blood cells (*10^3^/µL)	5.74 ± 1.23	5.41 ± 1.49	5.30 ± 1.69
Lymphocytes (*10^3^/µL)	2.31 ± 0.50	1.76 ± 0.54*	1.56 ± 0.40*
Monocytes (*10^3^/µL)	0.43 ± 0.09	0.43 ± 0.11	0.41 ± 0.11
Red blood cells (*10^6^/µL)	4.86 ± 0.57	4.62 ± 0.20	4.50 ± 0.24
Hemoglobin, g/dL	14.90 ± 1.61	14.58 ± 1.01	14.55 ± 0.73
Hematocrit, %	43.68 ± 4.66	42.19 ± 2.79	42.85 ± 1.85
Platelets (*10^3^/µL)	232.28 ± 32.20	243.18 ± 74.48	230.53 ± 51.04
Moderate activity, min/wk^a^	67 ± 66	315 ± 366	309 ± 357
Vigorous activity, min/wk^a^	44 ± 45	343 ± 106*	253 ± 109*
Training diary^b^			
Running, min/wk	–	360 ± 87	276 ± 134
Cycling, min/wk	–	–	184 ± 92
Swim, min/wk	–	–	119 ± 83
Resistance training, min/wk	–	119 ± 44	165 ± 30

Data are means ± standard deviation (SD). BMI, body mass index; WC, waist circumference; SBP, systolic blood pressure; DBP, diastolic blood pressure; OA, older active; RHR, resting heart rate; YA, young active; YA, young inactive. Superscripts indicate difference between groups determined using a one-way ANOVA with Tukey’s post-hoc testing. *p<0.05; **p<0.01 vs. YI and YA. ^a^Weekly physical activity minutes derived from the international physical activity questionnaire (IPAQ); ^b^detailed weekly training diaries were obtained from a subset (n=7–10) of YA and OA.

### Basal IL-10/6 receptor expression

**Figure 2:**
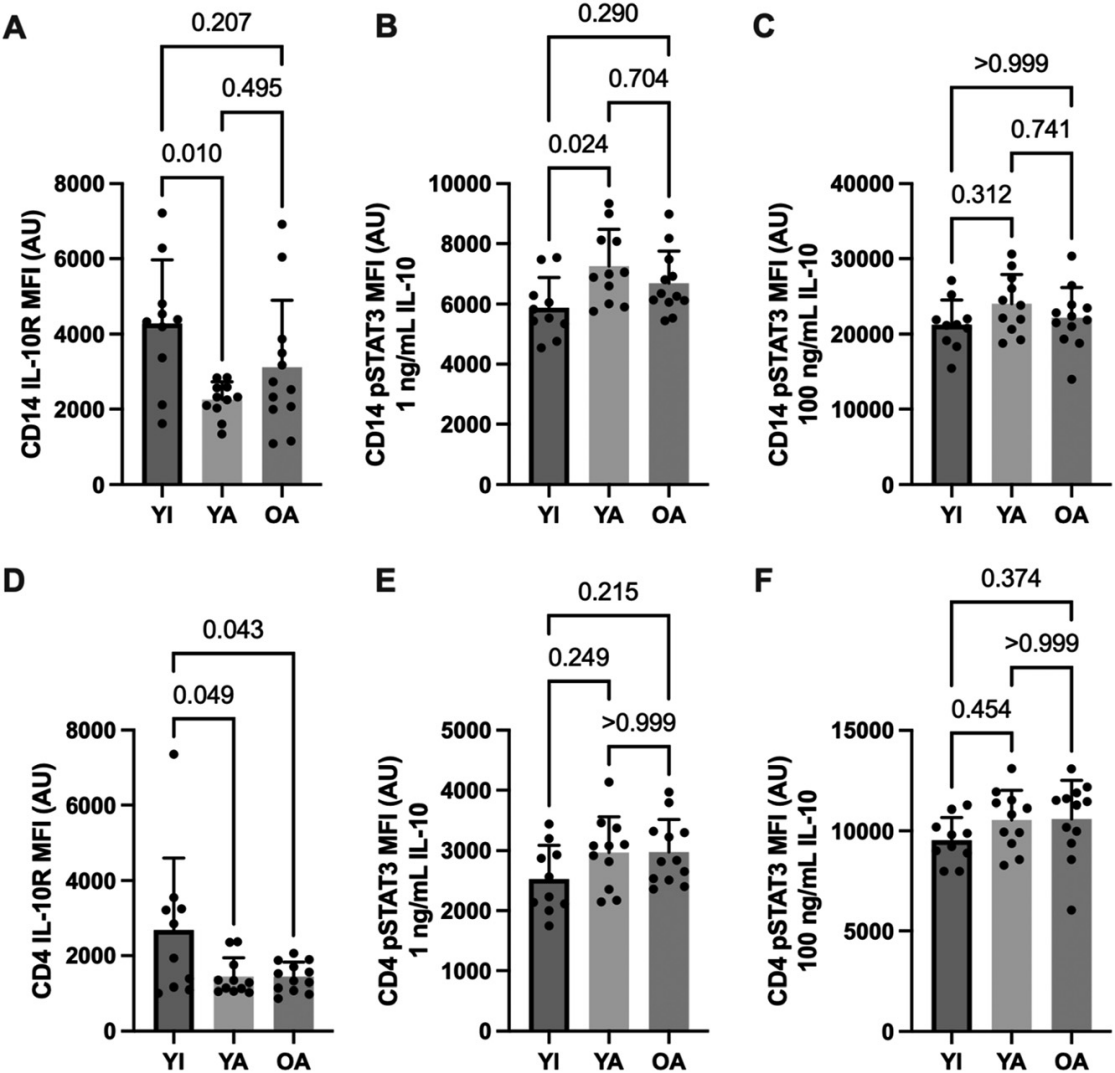
IL-10 receptor expression and IL-10 mediated STAT3 phosphorylation in CD14+ monocytes (A–C) and CD4+ lymphocytes (D–F). Data are shown as means ± SD and were analyzed using a one-way ANOVA. p-values shown above bars correspond to Tukey’s post-hoc testing for pairwise comparisons.

**Figure 3:**
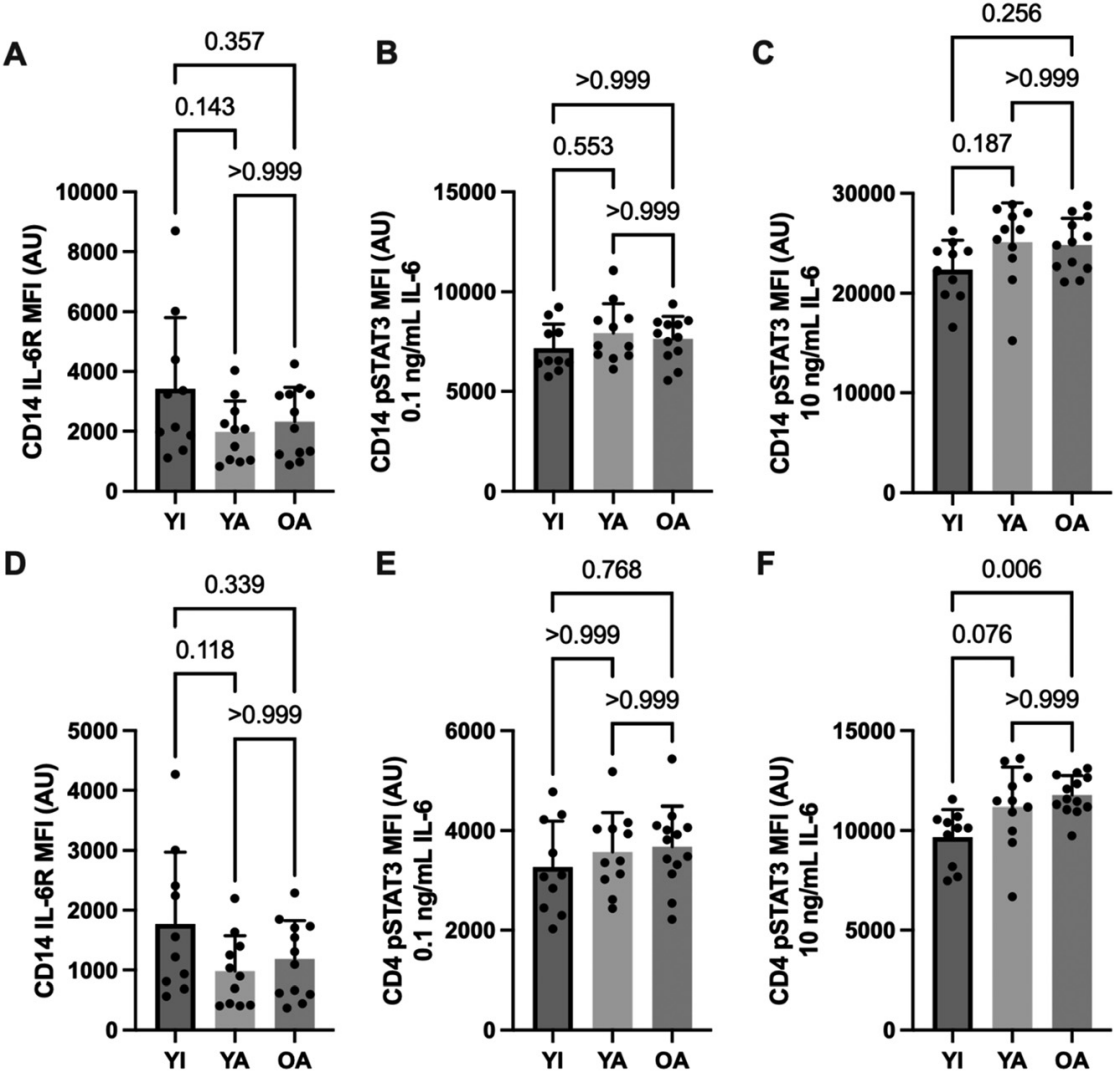
IL-6 receptor expression and IL-6 mediated STAT3 phosphorylation in CD14+ monocytes (A–C) and CD4+ lymphocytes (D–F). Data are shown as means ± SD and were analyzed using a one-way ANOVA. p-values shown above bars correspond to Tukey’s post-hoc testing for pairwise comparisons.

Basal (unstimulated) cytokine receptor expression in CD14 monocytes (panel A) and CD4 lymphocytes (D) are shown in Figure 2 (IL-10) and Figure 3 (IL-6). No between-group differences were apparent for unstimulated (basal) pSTAT3 MFI in either cell type (data not shown, p>0.48). IL-10 receptor expression was reduced in CD14 monocytes (p<0.01) and CD4 lymphocytes (p=0.05) in YA compared to YI (p<0.01). The OA group also had significantly lower CD4 IL-10 receptor expression compared to the YI group (p<0.05).

### IL-10/6 – mediated STAT3 phosphorylation

Cytokine-stimulated STAT3 phosphorylation in CD14 monocytes (panels B and C) and CD4 lymphocytes (E and F) are shown in Figure 2 (IL-10) and Figure 3 (IL-6). Submaximal (1 ng/mL) IL-10 stimulation elicited higher CD14 monocyte STAT3 phosphorylation in the YA vs. the YI group (p=0.02). A similar pattern was apparent for CD4 lymphocytes without significant differences between groups (p>0.21). In contrast, STAT3 phosphorylation in response to maximal (10 ng/mL) IL-10 stimulation was similar between groups in both cell types (p>0.31). No significant between-group differences were apparent for IL-6 receptor expression (p>0.11) or IL-6 – mediated STAT3 phosphorylation on CD14 monocytes (p>0.18). However, CD4 STAT3 phosphorylation was significantly higher in the OA compared to the YI group following maximal IL-6 stimulation (p<0.01), with a similar tendency observed in the YA group (p=0.08).

### LPS stimulated TNF-⍺ secretion and its inhibition by IL-10/6

**Figure 4:**
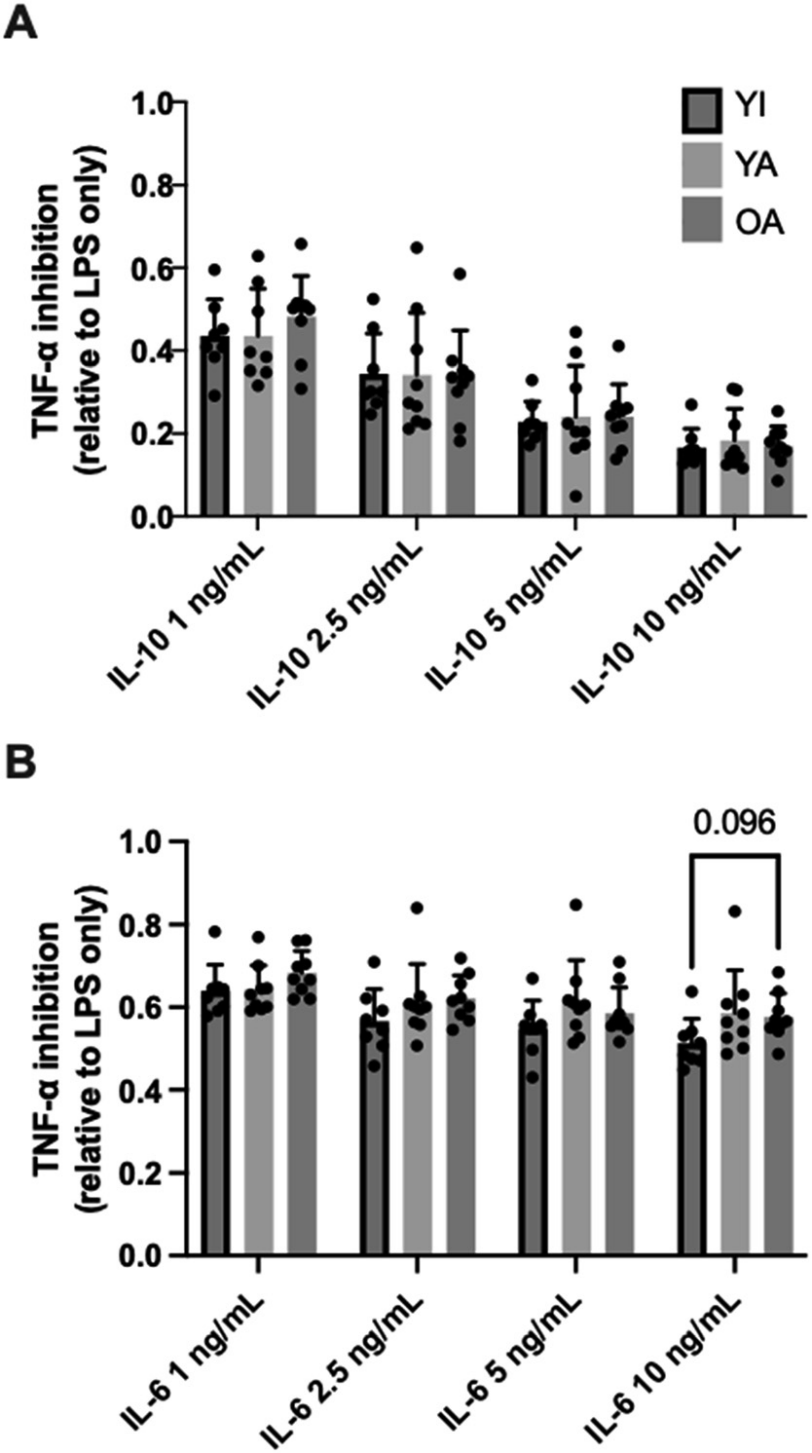
IL-10 (A) and IL-6 (B) mediated inhibition of TNF-⍺ secretion from LPS-stimulated whole-blood. Data are shown as means ± SD and were analyzed using mixed effects analysis. p-values shown above bars correspond to Tukey’s post-hoc testing for pairwise comparisons.

No significant between-group differences (p>0.71) were observed for LPS-stimulated TNF-⍺ secretion (YI: 314 ± 163 pg/mL; YA: 328 ± 101; OA: 365 ± 127). The ability of various concentrations of IL-10 and IL-6 to inhibit LPS-stimulated TNF-⍺ are shown in Figure 4A and B **,** respectively. Both cytokines inhibited TNF-⍺ secretion (main effect of concentration, p<0.01) though IL-10 was overall more anti-inflammatory eliciting a stronger dose-response inhibition of TNF-⍺ secretion. A significant group*concentration interaction effect (p=0.02) was observed with maximal IL-6 (10 ng/mL) co-treatment and appeared to be driven by a tendency for less TNF-⍺ inhibition in the OA vs. the YI group (p<0.10). No other differences for IL-10/6 mediated inhibition of TNF-⍺ secretion were apparent (p>0.05).

## Discussion

The purpose of this cross-sectional study was to compare blood leukocyte responsiveness to the anti-inflammatory cytokines IL-10 and IL-6 between individuals of varying activity levels and ages. The major finding was that the ability of IL-10 and IL-6 to activate STAT3 appeared to be enhanced in younger and older highly active individuals – despite lower leukocyte IL-10 receptor expression – when compared to younger inactive adults. These differences in leukocyte signaling were not associated with alterations in the ability of IL-10 or IL-6 to elicit decreases in the level of the pro-inflammatory cytokine TNF-⍺, which was similar across activity levels and preserved in older active individuals. These findings provide new insights into cellular cytokine action across age and physical activity status and support the anti-inflammatory potential of engaging in high levels of exercise/activity.

The impacts of exercise and training on systemic cytokines have been extensively studied but comparatively little is known about the associated impact on cytokine action at the cellular level [8]. This is important because cytokines typically function locally to impact cellular function after engaging with specific receptors and activating distinct signaling pathways [13]. We recently demonstrated that an acute bout of higher intensity exercise blunts the overall anti-inflammatory effects of IL-10/6 in active young adults [9] – a finding that appeared contradictory to the known anti-inflammatory benefits of regular physical activity [3, 4]. We speculated that this acute hyporesponsiveness to IL-10/6 action is likely transient in nature and confined to the immediate post-exercise period to enable inflammatory processes that facilitate cellular adaptations [14]. In this scenario, anti-inflammatory cytokine action would then return to or exceed pre-exercise levels, supporting an overall anti-inflammatory profile. Our current work partly supports this supposition by demonstrating that blood leukocytes from highly active individuals are indeed more responsive to IL-10/6 when studied under non-exercised (rested) conditions. Thus, despite the apparent transient hyporesponsiveness to anti-inflammatory cytokine action following each exercise bout [9], the ability of IL-10/6 to activate STAT3 signaling appears to be enhanced in highly active individuals compared to those who are inactive. The functional consequence of this greater STAT3 responsiveness remains to be determined, as the inhibition of TNF-⍺ secretion in response to LPS stimulation was similar between groups. Future work should therefore investigate addition events downstream of STAT3 signaling and/or cytokine secretion in response to other immune-activating stimuli to fully elucidate how anti-inflammatory cytokine action may be altered with chronically high levels of physical activity.

Intriguingly, the enhanced responsiveness to IL-10/6 in the highly active groups was apparent despite lower leukocyte IL-10 receptor expression (with a similar albeit non-significant pattern also observed for IL-6 receptor expression). Downregulation of the IL-10 receptor is likely an adaptive response to the high levels of physical activity in the YA/OA groups and the repeated stimulation of anti-inflammatory cytokines that is often reported following individual exercise bouts [4, 8]. The enhanced responsiveness of blood leukocytes to IL-10/6 is likely needed to allow activation of intracellular signaling pathways with fewer cytokine receptors – particularly since cytokine responses to acute exercise can sometimes be attenuated with training [15]. The enhanced sensitivity to IL-10 action could also be a contributor to the frequently purported immunosuppressive effects of very high levels of activity [16, 17]. Others have also reported associations between antigen stimulated IL-10 production and training load in athletes [18, 19]. The present data suggest a role of altered IL-10 action – in addition to production – in the potential immunosuppressive effects of high exercise volumes.

Although our study focused on the anti-inflammatory potential of IL-6 in the context of physical activity, this cytokine has known pro-inflammatory effects in other contexts that should be considered when interpreting the biological significance of IL-6 action [20, 21]. In our study, the IL-6 mediated activation of STAT3 was accompanied by inhibition of TNF-⍺ secretion from LPS-stimulated whole blood co-treated with IL-6, supporting an overall anti-inflammatory effect that is consistent with previous work 22], [23], [24. However, the inhibition of TNF-⍺ secretion by IL-6 was less pronounced than IL-10 – an observation that we have reported previously [10] – confirming that IL-6 is less anti-inflammatory than IL-10. Moreover, it is unclear if the inhibition of TNF-⍺ secretion in IL-6 treated whole-blood cultures was due to direct effects of IL-6 *per se* or via downstream stimulation of IL-10 secretion [23, 25]. Additional work is needed to discern the direct and indirect anti-inflammatory actions of IL-6 in blood leukocytes and how these may be impacted by physical activity.

Age-related alternations in immune function – often termed immunosenescence or inflammaging – are a proposed contributor to poor health and heightened chronic disease risk in older individuals [11, 26]. Although the accompanying reductions in physical activity make it difficult to disentangle the direct effects of aging *per se* from physical inactivity, exercise has known immunomodulatory benefits in older adults [11]. Our observation of greater STAT3 activation in IL-6 – stimulated blood leukocytes from older active individuals compared to their younger inactive counterparts provides an additional mechanism by which high levels of activity may help maintain an anti-inflammatory profile through the lifespan. Importantly, the ability of both IL-10 and IL-6 to inhibit the secretion of TNF-⍺ secretion was preserved in the older active adults in our study, suggesting that the ability of these cytokines to combat inflammation does not deteriorate through the middle age with high levels of physical activity. These observations are in line with studies examining plasma cytokine concentrations in older adults, which are also supportive of a more anti-inflammatory profile in those of higher physical activity levels [27].

The enhanced ability of IL-10/6 to activate intracellular signaling in monocytes and lymphocytes from active individuals may have implications for broader changes in immune function with physical activity. For instance, it is well-established that chronically high levels of physical activity lower the risk of mortality and morbidity via reduced chronic inflammation (e.g., via lowered expression and/or activation of toll-like receptors on innate immune cells) [3]. The ability of physical activity to modulate responsiveness of innate immune cells such as monocytes to IL-10 stimulation could be a contributor to the inflammation-lowering effects of physical activity. On the other hand, a heightened responsiveness of monocyte signaling to IL-10 could also contribute to tempered innate immune responses in highly active individuals, making them more susceptible to immunosuppression and potentially contributing to the elevated rates of respiratory infections that are sometime reported in athletes [16, 17]. Given the role of IL-6 in regulation of T cell differentiation and effector functions, the greater responsiveness of CD4 cells to IL-6 in more active individuals could also be associated with altered innate immunity [28]. Future research including additional immune cell subtypes, immune activating stimuli, and/or the assessment of immune cell effector responses is needed to further elucidate how physical activity alters the regulation of innate and adaptions immune functions by IL-10/6.

The current study had some noteworthy strength and limitations. First, we directly measured cytokine action by quantifying the ability of various concentrations of IL-10/6 to activate STAT3 signaling in distinct blood leukocyte populations and inhibit TNF-⍺ secretion *ex vivo.* Although we recognize the small sample size of the current study, we matched the number of males and females within each group to equalize the potential impact of biological sex, which we have previously shown to be a modulator of anti-inflammatory cytokine action [12]. We also recognize that due to the lack of an inactive older group, the direct effects of aging *per se* on cytokine action could not be disentangled from physical activity providing an important next step for future work in the area. Although comorbidities and medications can often make age-related comparisons difficult, future studies should also seek to explore anti-inflammatory cytokine action in older age groups.

## Conclusions

Blood leukocytes from active individuals exhibit more responsiveness to IL-10/6 stimulation – as reflected by greater cytokine stimulated STAT3 phosphorylation *ex vivo* – compared to individuals who are physically inactive. The heightened ability of these anti-inflammatory cytokines to activate STAT3 was apparent despite lower cytokine receptor expression, pointing to enhanced responsiveness of intracellular signaling with high levels of activity. The ability of IL-10/6 to inhibit pro-inflammatory cytokine secretion was not impacted by physical activity level, but was preserved in older active individuals compared to their younger counterparts.
